# Editorial: Advancements in the Understanding of Anthropogenic Impacts on the Microbial Ecology and Function of Aquatic Environments

**DOI:** 10.3389/fmicb.2021.820697

**Published:** 2022-01-24

**Authors:** Rodrigo G. Taketani, Francisco Dini-Andreote, Sara Beier, Camila Fernandez

**Affiliations:** ^1^Sustainable Agriculture Sciences. Rothamsted Research, Harpenden, United Kingdom; ^2^Department of Plant Science & Huck Institutes of the Life Sciences, The Pennsylvania State University, University Park, PA, United States; ^3^Department of Biological Oceanography, Leibniz Institute for Baltic Sea Research, Warnemünde, Germany; ^4^LOMIC UMR7621 CNRS Observatoire Oceanologique de Banyuls sur Mer, Languedoc-Roussillon, France; ^5^COPAS SUR AUSTRAL, COPAS COASTAL, INCAR Center, Universidad de Concepcion, Concepción, Chile; ^6^IDEAL Center, Universidad Austral, Valdivia, Chile

**Keywords:** microbial diversity, anthropogenic impact, bioremediation, biodiversity, global climate change

Aquatic environments are important ecosystems providing multiple services to humankind and directly affecting economic income worldwide. These ecosystems have increasingly been threatened by changes in global climate and diverse anthropogenic activities—from agriculture to industry (Häder et al., 2020). In general, the continuous exploitation of aquatic ecosystems has caused severe impacts on biological diversity despite some efforts to control habitat exploitation via local legislation (Popper et al., 2020). For instance, the leaching of pollutants and chemical hazards, the eutrophisation caused by extensive use of chemicals in agriculture and aquaculture, changes in land use, and the disposal of urban wastes; are the major factors responsible for most of the anthropogenic impacts on these ecosystems worldwide (Cotta et al., 2019). As such, properly monitoring the effects of these human activities is critical to aid the early detection of potential chemicals and activities with large impacts in aquatic ecosystems. Besides, advances in ecological research can provide the basis for developing new strategies of remediation and recovery of impacted systems (Taketani et al., 2010).

Microbial organisms are highly abundant, ubiquitous, and main drivers of all biochemical cycles (Falkowski et al., 2008), which makes them important players of ecosystem services in all systems, including aquatic environments (Cavicchioli et al., 2019). Due to the sensitivity of microbial community dynamics to any kind of environmental change (Shade et al., 2012), microbes may further serve as indicator species for detecting anthropogenic activities in natural environments (Parmar et al., 2016).

The goal of this research topic was to collect studies that helped to understand the impacts of human presence and activities on the microbial ecology of aquatic ecosystems, and to harness microbial metabolisms to remediate impacted areas. This topic contains a series of 12 articles that cover a wide range of aquatic systems, including lakes, corals, estuaries, and marine waters. We were fortunate to receive research articles in systems across the globe, from North and South America, Africa, Oceania, Asia, and Europe, covering also variation in biogeography and climate conditions.

Out of the 12 articles published in this topic, three manuscripts focused on different aspects of the coral holobiont. Wambua et al. showed the effect of different levels of anthropogenic impacts on Kenyan corals using metagenome analysis. The results indicate that corals from sites under relatively higher anthropogenic activities were enriched in genes associated with a reduction in intracellular levels of environmental contaminants and DNA repair. Moreover, Ezzat et al. evaluated the effect of elevated water temperature and sturgenfish fecal pellet on the microbiome of the coral *Porites lobata*. This study revealed an increase in the abundance of some coral pathogenic taxa and linked the presence of feces with the inability of corals to recover from wounds. Thus, it suggests that the interaction between higher temperatures and feces are detrimental to coral health. Last, Assis et al. studied the use of rotifers as a delivery system of Beneficial Microorganisms for Corals (BMCs) to improve coral health. This research article nicely illustrates that corals are capable of uptaking the rotifer carrying BMC probiotic, as visualized by electron microscopy. Together, these articles cover various important aspects on the issue of anthropogenic impacts in coral systems that range from detecting the nature and magnitude of the impact toward understanding the ecological effect, and proposing methods for remediation.

Chen et al. assessed how different environmental factors influence surface water *Vibrio* populations from seven seasonally sampled sites located in the Maowei Sea, China. These authors showed that factors related to eutrophication (e.g., total dissolved N and total dissolved P) are key determinants for the selection of different *Vibrio* taxa. Of critical importance, this was argued to have a direct impact on the health of the surrounding human population. In another article, Abioye et al. assessed the prevalence of a set of medically important *Vibrio* species in South African water sources. Most interestingly, this study revealed the presence of Vibrio spp. in a higher number of tested samples, and this fact was later associated with the pollution level of these sources. Both articles highlight the importance of monitoring the presence of pathogenic organisms in water systems, and link their presence with levels and types of chemical pollution.

This research topic also includes three articles focusing on aquaculture. Valdés-Castro and Fernandez studied the effect of aquaculture pesticides on the natural microbial community in the coast of Chile. The authors showed the response of microbial communities to the addition of pesticides to be variable; i.e., either stimulating or inhibiting microbial activity, but with a potentially important and consistent impact on nitrogen budget in the system. Wang et al. studied the effect of different management practices in crayfish production. This study illustrates how different practices exert intense impacts on microbial communities in the system, and discuss strategies for the development of better practices. Fieler et al. investigated seasonal variation in coastal waters off Norway in kelp production through the seasons assessing the kelp matter balance. The obtained results indicate that the time of harvest was a key factor influencing carbon fixation in the system and balance in kelp production. Together, these articles show the importance of informed management practices and their idiosyncrasies across different aquaculture systems.

Two research papers also investigated the impact of xenobiotics on aquatic microbial communities. Kamalanathan et al. used mesocosms to investigate the short- and long-term effects of oil exposure following the Macondo oil spill on microbial diversity and function. In brief, this study describes a disruption of the co-occurrence pattern in microbial taxa due to oil exposure. This was suggested to have a direct impact on community composition, the ecology of biological interactions between taxa, and the functions they perform in the system. Janßen et al. developed a method using Machine Learning to analyse microbial signatures in community composition, and to detect 2,4,6-trinitrotoluene (TNT) in southwestern Baltic Sea sediments via microbial community fingerprints. The use of this method nicely predicted the presence of TNT with 81.5% balanced accuracy, even though the presence of this compound was not *per se* a significant driver of community divergences.

Li et al. studied differences in microbial communities in freshwater lakes occurring at different elevations. This study shows a higher prevalence of genes related to environmental stresses in microbial communities inhabiting lakes at higher elevations. Liu et al. investigated the influence of environmental factors on the community assembly among subalpine lakes. They observed that the community was influenced by carbon content and spatial distance.

Taken together, the studies presented in this research topic cover a wide variety of issues related to anthropogenic activity from aquaculture to global climate change including the presence of xenobiotics and other disturbances. These articles highlight the greater importance of microorganisms as pivotal biotic factors directly impacted by these practices and the most important agents to be properly exploited for ecosystem remediation in affected areas. In summary, articles in this Frontiers research topic shed light on the continuous negative and potentially deleterious effects of human action on aquatic ecosystems. We hope that this collection of papers will be a valuable resource for researchers and stakeholders and will enhance the role of microbial ecology as a foundational basis toward improving research and public policy. We ultimately hope to contribute to promoting advances in sustainability and monitoring anthropogenic impacts on aquatic ecosystems.

## Author Contributions

All authors of this editorial article have contributed in the conceptualization, writing, reviewing, revising, and have approved it for publication.

## Conflict of Interest

The authors declare that the research was conducted in the absence of any commercial or financial relationships that could be construed as a potential conflict of interest.

## Publisher's Note

All claims expressed in this article are solely those of the authors and do not necessarily represent those of their affiliated organizations, or those of the publisher, the editors and the reviewers. Any product that may be evaluated in this article, or claim that may be made by its manufacturer, is not guaranteed or endorsed by the publisher.

## References

[B1] Cavicchioli R. Ripple W. J. Timmis K. N. Azam F. Bakken L. R. Baylis M.. (2019). Scientists' warning to humanity: microorganisms and climate change. Nat. Rev. Microbiol. 17, 569–586. 10.1038/s41579-019-0222-5 31213707 PMC7136171

[B2] Cotta S. R. Cadete L. L. van Elsas J. D. Andreote F. D. Dias A. C. F. (2019). Exploring bacterial functionality in mangrove sediments and its capability to overcome anthropogenic activity. Mar. Pollut. Bull. 141, 586–594. 10.1016/j.marpolbul.2019.03.001 30955771

[B3] Falkowski P. G. Fenchel T. Delong E. F. (2008). The microbial engines that drive Earth's biogeochemical cycles. Science 320, 1034–1039. 10.1126/science.1153213 18497287

[B4] Häder D.-P. Banaszak A. T. Villafañe V. E. Narvarte M. A. González R. A. Helbling E. W. (2020). Anthropogenic pollution of aquatic ecosystems: emerging problems with global implications. Sci. Total Environ. 713:136586. 10.1016/j.scitotenv.2020.136586 31955090

[B5] Parmar T. K. Rawtani D. Agrawal Y. K. (2016). Bioindicators: the natural indicator of environmental pollution. Front. Life Sci. 9, 110–118. 10.1080/21553769.2016.1162753

[B6] Popper A. N. Hawkins A. D. Thomsen F. (2020). Taking the animals' perspective regarding anthropogenic underwater sound. Trends Ecol. Evol. 35, 787–794. 10.1016/j.tree.2020.05.002 32466956

[B7] Shade A. Peter H. Allison S. D. Baho D. L. Berga M. Bürgmann H.. (2012). Fundamentals of microbial community resistance and resilience. Front. Microbio. 3:417. 10.3389/fmicb.2012.00417 23267351 PMC3525951

[B8] Taketani R. G. Franco N. O. Rosado A. S. van Elsas J. D. Elsas J. D. (2010). Microbial community response to a simulated hydrocarbon spill in mangrove sediments. J. Microbiol. 48, 7–15. 10.1007/s12275-009-0147-1 20221723

